# 2-Oxoglutarate-dependent dioxygenases in the biosynthesis of simple coumarins

**DOI:** 10.3389/fpls.2014.00549

**Published:** 2014-11-03

**Authors:** Bun-Ichi Shimizu

**Affiliations:** Department of Life Sciences, Graduate School of Life Sciences, Toyo UniversityItakura, Japan

**Keywords:** coumarin biosynthesis, simple coumarins, *Ortho*-hydroxylases, coenzyme A thioester of cinnamates, C-terminal sequences, Arabidopsis, *Ipomoea batatas*, *Ruta graveolens*

## Abstract

Coumarins are natural plant products that have been the subject of extensive phytochemical and pharmacological research studies in the past few decades. The core structure of coumarins is derived from the respective cinnamates via *ortho*-hydroxylation of the aromatic ring, *trans/cis* isomerization, and lactonization. Various substitution patterns of coumarins have been reported, whereas the biosynthesis of coumarins remains elusive. *Ortho*-hydroxylation is a key step in simple coumarin biosynthesis as a branch point from the lignin biosynthetic pathway. 2-Oxoglutarate-dependent dioxygenases (2OGDs) from plants convert cinnamate derivatives into simple coumarins through the process of *ortho*-hydroxylation. This review describes the 2OGDs involved in coumarin biosynthesis and their substrate specificities.

## Introduction

**Figure 1:**
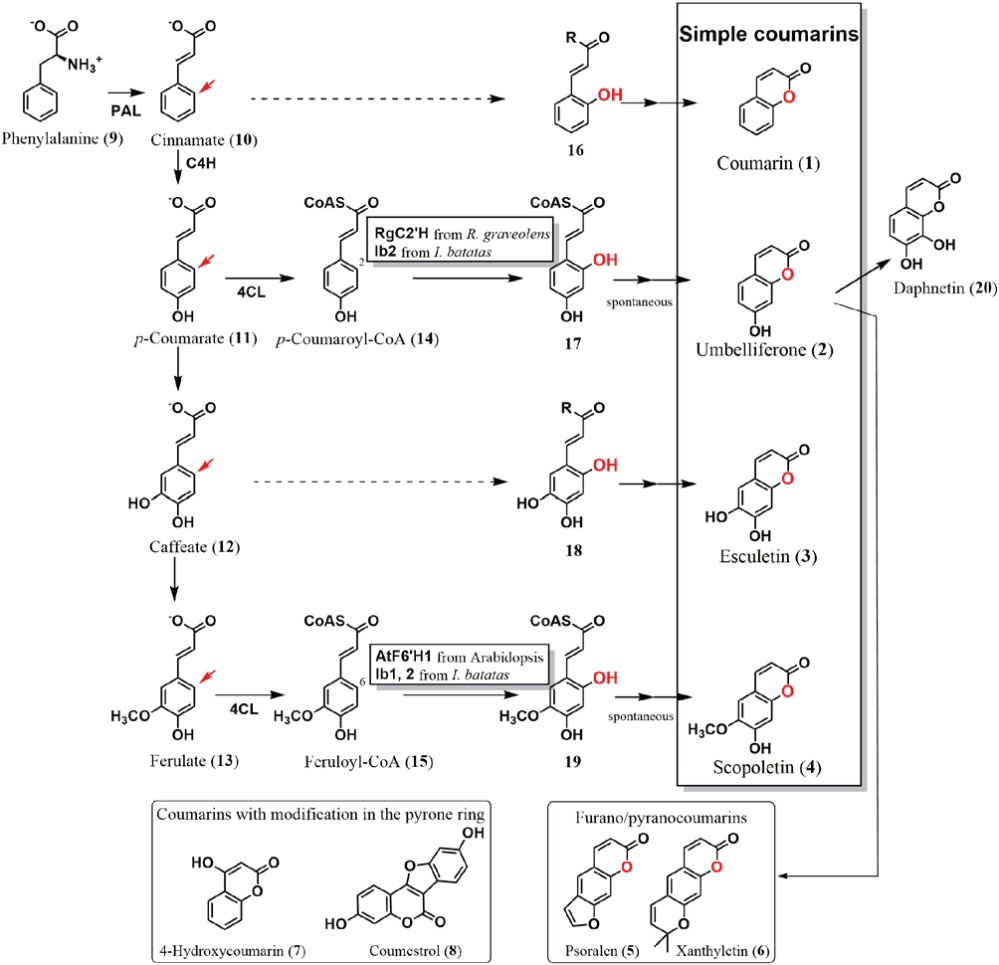
**Coumarin biosynthetic pathway in plants**. Simple coumarins, coumarin (**1**), umbelliferone (**2**), esculetin (**3**), and scopoletin (**4**) have modifications in their benzene ring. They are biosynthesized from the phenylpropanoid pathway via *ortho*-hydroxylation of cinnamate (**10**), *p*-coumarate (**11**), caffeate (**12**), and ferulate (**13**), respectively. The *ortho*-positions are shown by red arrows. Oxygen atoms introduced by *ortho*-hydroxylation are also highlighted in red. The *ortho*-hydroxylases from Arabidopsis (AtF6′H1), *Ruta graveolens* (RgC2′H), and *Ipomoea batatas* (Ib1 and Ib2) were functionally analyzed. AtF6′H1 and Ib1 catalyze *ortho*-hydroxylation of feruloyl-CoA (**15**), whereas RgC2′H and Ib2 were capable of reacting to both feruloyl-CoA (**15**) and *p*-coumaroyl-CoA (**14**) as the substrates. After hydroxylation, *trans*/*cis* isomerization and lactonization occur, resulting in the production of their respective coumarins. Umbelliferone (**2**) is a key intermediate of prenylcoumarin biosynthesis, from which furanocoumarins and pyranocoumarins (examples: psoralen and xanthyletin, respectively) are derived. No report has described cloning and functional analysis of the hydroxylases that introduce an *ortho*-hydroxy group to cinnamate and caffeate to form coumarin (**1**) and esculetin (**3**), respectively (hashed arrows). Coumarins substituted in the pyrone ring are thought to be derived from different pathways.

Coumarins are common plant-derived natural products that are characterized by its core structure, coumarin (**1**, Figure 1). These molecules exhibit various biological activities such as antibacterial (Schinkovitz et al., 2003; Stavri et al., 2003; Céspedes et al., 2006), antioxidant (Bajerova et al., 2014), anti-inflammatory (Witaicenis et al., 2013), rodenticidal (Lotfi et al., 1996), termiticidal (Adfa et al., 2010, 2011), and other activities (Stahmann et al., 1941; Murray, 1989; Runkel et al., 1996; Song et al., 2014). In addition, the role(s) of coumarins in plants have also been reported. Scopoletin in tobacco is accumulated during a hypersensitive response (Gachon et al., 2004) and is considered to be involved in virus resistance (Chong et al., 2002). In *Arabidopsis thaliana*, coumarins play a role as a chelator of iron ions in soil (Fourcroy et al., 2013; Schmid et al., 2013; Schmidt et al., 2014).

Based on their structural and biosynthetic properties, plant coumarins are categorized as follows: simple coumarins, furanocoumarins, and pyranocoumarins, and coumarins with modifications in the pyrone ring (Figure 1) (Keating and O'kennedy, 1997). Simple coumarins harbor the hydroxy (-OH), alkoxy (-OR), and/or alkyl (-R) group(s) in their benzene ring: coumarin (**1**), umbelliferone (**2**: 7-hydroxycoumarin), esculetin (**3**: 6,7-dihydroxycoumarin), and scopoletin (**4:** 7-hydroxy-6-methoxycoumarin). Their hydroxy group is involved in conjugation to produce glycosides (Tal and Robeson, 1986; Taguchi et al., 2000, 2001; Shimizu et al., 2005; Kai et al., 2006; Bayoumi et al., 2008b; Wu et al., 2009). Furanocoumarins and pyranocoumarins have additional ring systems, a five–or six-memberd ring with an oxygen atom, which are fused to the benzene ring.

Plant researchers consider coumarins as a potential fluorescent and flavoring component (Poulton et al., 1980; Oba et al., 1981; Mock et al., 1999; Katerinopoulos, 2004; Bourgaud et al., 2006; Stanfill et al., 2007; Maggi et al., 2011; Krieger et al., 2013). Tracer experiments using cinnamate (**10**) or its derivatives have effectively shown that simple coumarin formation in plants proceeds via hydroxylation of the *ortho*-position (*ortho*-hydroxylation) of respective cinnamates, the adjacent position in the benzene ring to the side chain (Brown et al., 1960; Brown, 1962; Fritig et al., 1970; Bayoumi et al., 2008a), followed by formation of a lactone ring. Furanocoumarins and pyranocoumarins are derived from umbelliferone (**2**) by addition of prenyl group (Larbat et al., 2007; Karamat et al., 2013). 4-Hydroxycoumarin (**7**) in Apiaceae and Asteraceae plants is presumed to utilize another biosynthetic pathway that does not require *ortho*-hydroxylation (Liu et al., 2009). It has been previously suggested that coumestrol (**8**) in Leguminosae plants, which also comprises a coumarin core structure, is synthesized from isoflavonoids, circumventing the need for *ortho*-hydroxylation of cinnamates in its biosynthetic pathway (Veitch, 2013).

Due to its irreversibility, *ortho*-hydroxylation is considered a key step in the biosynthesis of simple coumarins. This review summarizes the research findings on *ortho*-hydroxylation enzymes (*ortho*-hydroxylases) of cinnamates that are involved in simple coumarin biosynthesis. The distribution of the *ortho*-hydroxylases in plants using a database search of EST homologs will be also discussed.

## 2-oxoglutarate-dependent dioxygenases involved in the *ortho*-hydroxylation of cinnamates are the key enzymes of simple coumarin biosynthesis

In Arabidopsis, a 2-oxoglutarate-dependent dioxygenase (2OGD) encoded by the gene *AtF6′H1* (locus: At3g13610) was found to exhibit *ortho*-hydroxylase activity to feruloyl coenzyme A (**15**: feruloyl-CoA) as a substrate, with a *K*_*m*_ value of 36 μM, yielding an *ortho*-hydroxylation product, 6-hydroxyferuloyl-CoA (**19**) (Kai et al., 2008). The AtF6′H1 enzyme exhibits no catalytic activity to *p*-coumaroyl-CoA (**14**), free ferulic acid (**13**), or feruloyl quinate. Deficient mutation of the *AtF6′H1* gene in *Arabidopsis* causes a significant reduction in the accumulation of scopolin, a β-glucoside of scopoletin (**4**), indicating that AtF6′H1 catalyzes *ortho*-hydroxylation. Another 2OGD (AtF6′H2) encoded by a homologous gene (locus: At1g55290) exhibits an equivalent activity against CoA thioesters of cinnamates (*K*_*m*_ value for feruloyl-CoA: 14.5 μM); however, no significant change in scopolin levels was observed in the plant.

Further studies involving cloning and functional analysis of the 2OGD genes in plants have elucidated the mechanism of coumarin formation. Using *Ruta graveolenes*, which accumulates franocoumarins, a 2OGD (RgC2′H) was cloned as the key enzyme of coumarin biosynthesis (Vialart et al., 2011). RgC2′H shows hydroxylation activity not only to feruloyl-CoA (**15**, *K*_*m*_ = 37 μM), but also to *p*-coumaroyl-CoA (**14**, *K*_*m*_ = 50 μM), forming scopoletin (**4**) and umbelliferone (**2**), respectively. Furanocoumarins are formed after addition of prenyl group to umbelliferone (**2**), which is detected in *R. graveolens*, whereas no scopoletin (**4**) was detected. This result indicates that RgC2′H exclusively catalyzes *p*-coumaroyl-CoA (**14**), besides its activity against feruloyl-CoA (**15**) and *p*-coumaroyl-CoA (**14**). Regulation of substrate supply to RgC2′H enzyme is likely to determine the structures of the products, namely, umbelliferone (**2**) or scopoletin (**4**).

The biosynthetic origin of the 1-oxygen atom of umbelliferone (**2**) in sweet potato root (*Ipomoea batatas*) is molecular oxygen; therefore, hydroxylase using a water molecule to introduce a hydroxy group was excluded as the candidate of *ortho*-hydroxylation enzyme(s) (Shimizu et al., 2008). 2OGDs from sweet potato were also cloned and functionally analyzed as the *ortho*-hydroxylases of CoA thioesters of the cinnamates (Matsumoto et al., 2011). The 2OGDs were then categorized into two groups based on their substrate specificities. Enzymes belonging to the first one, designated as Ib1s, showed *ortho*-hydroxylation activity to feruloyl-CoA (**15**, *K*_*m*_ = approximately 10 μM), whereas those of Ib2s catalyzed both *p*-coumaroyl-CoA (**14**, *K*_*m*_ = 7.3–14 μM) and feruloyl-CoA (**15**, *K*_*m*_ = 6.1–15.2 μM) as the substrates to yield umbelliferone (**2**) and scopoletin (**4**), respectively. Root tissues of sweet potato accumulate moderate levels of scopolin. After fungal and elicitor treatments, the production of umbelliferone (**2**) and its β-glucoside, skimmin, was significantly higher than that before treatment, whereas the amount of scopolin remained at a moderate level after the treatments. Fungal and elicitor treatments also resulted in an upregulation of *Ib2* genes, whereas no significant induction of *Ib1* genes was detected. These results indicate that Ib2s mainly synthesize umbelliferone (**2**) using *p*-coumaroyl-CoA (**14**), besides their bi-functional activity.

In *R. graveolens* and *I. batatas*, the *ortho*-hydroxylases may act as neighboring enzymes by positioning themselves at enzymes of the upper steps such as C4H, C3H, or 4CL, and receive more supplies with their substrate, *p*-coumaroyl-CoA (**14**), to produce umbelliferone (**2**). Interactions among the metabolic enzymes (Burbulis and Winkel-Shirdley, 1999) including the *ortho*-hydroxylases possibly occur when simple coumarins are biosynthesized in these plant cells.

The *ortho*-hydroxylase involved in the formation of coumarin (**1**) is still unknown, whereas approaches to biosynthesis of coumarin (**1**) have been performed using sweet clover (Gestetner and Conn, 1974) and lavender (Brown et al., 1960; Stoker and Bellis, 1962). Esculetin (**3**) formation is also remained to be elucidated. Ib1s from sweet potato showed a trace activity to caffeoyl-CoA (Matsumoto et al., 2011). Therefore, catalysis of these reactions by members of the 2OGD family is reasonable using cinnamate (**10**) or caffeate (**12**) esters, or their free acid, respectively. Enzymatic information of *ortho*-hydroxylase homologs would tell mechanism of these coumarins. There is still a possibility that other enzyme families such as flavin monooxygenases or another oxidase family would also contribute to this reaction (Schlaich, 2007). Furthermore, in cassava or chicory, modification steps involving the conversion of umbelliferone (**2**) to esculetin (**3**) or daphnetin (**20**: 7,8-dihydroxycoumarin) have been detected by tracer analysis, indicating a biosynthetic grid of simple coumarin formation (Sato and Hasegawa, 1972; Bayoumi et al., 2008a).

Although the details of the biosynthesis of simple coumarins are still unclear, the three examples of *ortho*-hydroxylases serve as key information for future researches on elucidating the mechanism of coumarin biosynthesis in plants. Substrate specificities of the *ortho*-hydroxylases from plants that accumulate coumarins will be also clue to know the metabolic grid of coumarin biosynthesis.

## Quest for the candidate sequences of *ortho*-hydroxylases in plants

**Figure 2:**
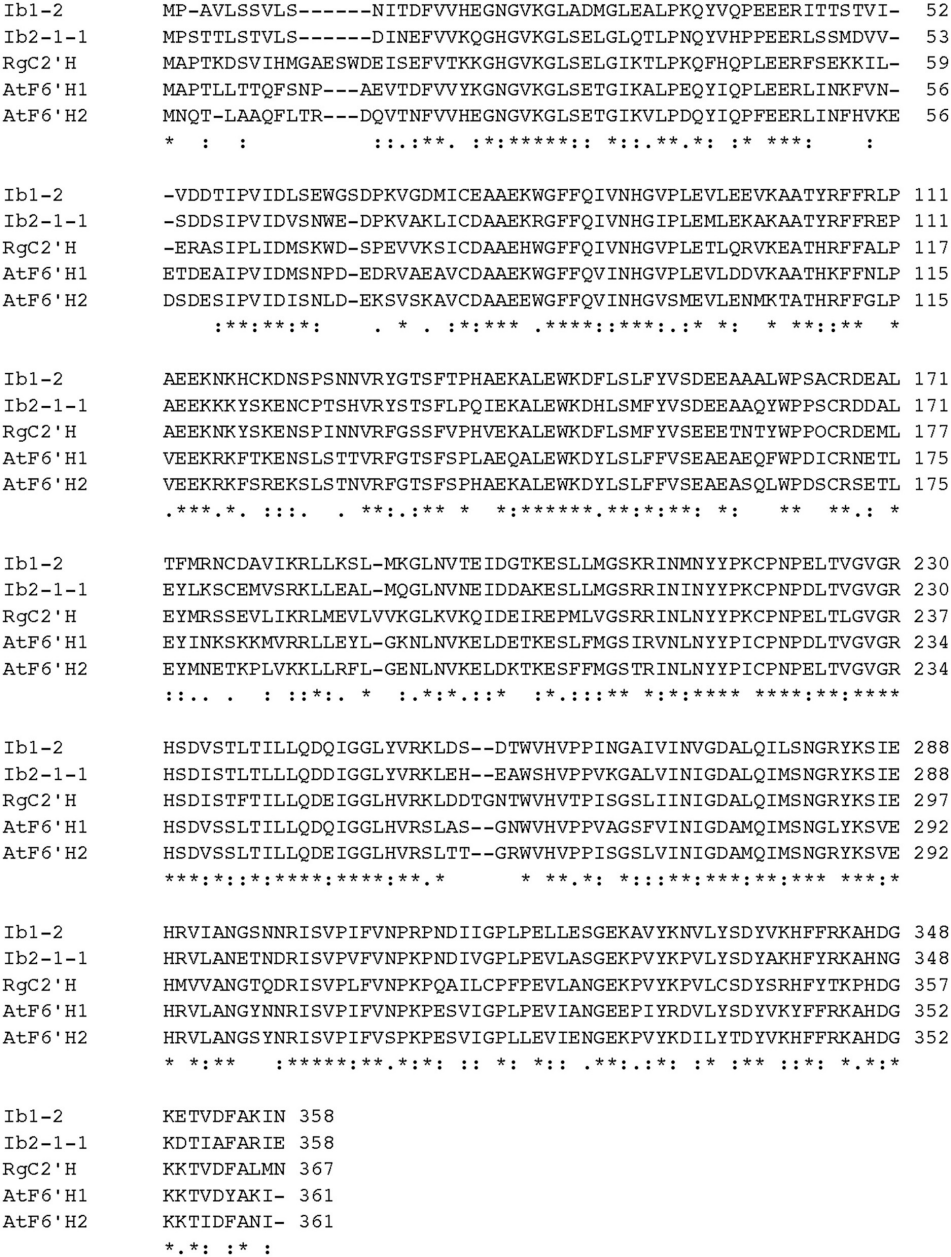
**Comparison of amino acid sequences of *ortho*-hydroxylases from the plants**. Amino acid sequences are aligned using ClustalW2 (McWilliam et al., 2013, http://www.ebi.ac.uk/Tools/msa/clustalw2/). A FASTA file of the protein sequences is available as Supplementary Material 3.

The substitution patterns involving the phenyl group of cinnamates have been extensively characterized. Furthermore, the CoA moiety is a prerequisite for their activity. The alignment of the amino acid sequences of previously reported *ortho*-hydroxylases is presented in Figure 2, which shows a moderately high sequence identity (approximately 59–64% amino acid identity), with conserved amino acid residues. Investigation of substrate specificities of 2OGDs using chimeric proteins revealed the significance of C-terminal sequence elements of gibberellin 20-oxidases of *Cucurbita maxima* (Lange et al., 1997) and flavanone 3β-hydroxylase of *Petunia* sp. (Wellmann et al., 2004). They reported that the C-terminal sequences comprising 33–54 amino acid residues are involved in substrate recognition.

Taking advantage of these results, a TBLASTN search (http://blast.ncbi.nlm.nih.gov/Blast.cgi; Altschul et al., 1997) was performed to explore candidate EST sequences of *ortho*-hydroxylases involved in the biosynthesis of simple coumarins, using the C-terminal sequences of AtF6′H1 (54 amino acid residues, Supplementary Material 1).

The results (maximum target sequences: 1000; Supplementary Material 2) showed that the hit sequences belonged to the 2OGD family, with maximum scores within the range of 42–111 and minimum *E*-values within the range of 1 e^−27^–1 e^−2^. The highest scoring hits were observed in the Brassicales plants. Although it was necessary to analyze the accumulation of simple coumarins, these clones would show *ortho*-hydroxylase activity, thus indicating its involvement in simple coumarin formation. Plant species belonged to Spindales, Malvales, Malpigiales, Fabales, Rosales, Fagales, Vitales, Solanales, Lamiales, Gentianales, and Asteriales also showed significantly high scores and low *E*-values, whereas other plant species with 2OGD sequences were of relatively lower levels of similarity. In plants that accumulate simple coumarins, 2OGDs with higher levels of similarity are likely to exhibit *ortho*-hydroxylase activity. In Fabales, *Lotus japonicus*, *Glycine max*, *Vigna unguiculata*, and *Medicago truncatula* harbored ESTs with highly similar sequences. Coumarin is accumulated in *Melilotus alba*, a Fabales plant (Brown et al., 1960; Stoker and Bellis, 1962; Gestetner and Conn, 1974). These EST sequences in Fabales plants could serve as clues in the search for *ortho*-hydroxylases in cinnamate (**10**) from *M. alba*. In addition, sequences from *Euphorbia* spp. or *Manihot esculenta*, which accumulate esculetin (Masamoto et al., 2003; Bayoumi et al., 2008a; Nazemiyeh et al., 2009; Shi et al., 2009), showed high similarities. The biosynthetic pathway of simple coumarins containing esculetin in these plants would be elucidated through the functional analysis of these sequences. Species from the rest of the orders were less similar to the partial sequence of AtF6′H1.

Kawai et al. (2014) conducted an extensive phylogenetic analysis of 2OGD sequences, where the *ortho*-hydroxylases involved in simple coumarin biosynthesis belonged to DOXC30-clade. These enzymes were not detected in *Oryza sativa* or other vascular plants that arose from more basal lineages (Stevens, 2014). There is no report about coumarin accumulation in *O. sativa*. The tendency decrease in the level of similarity in the EST sequences supports the results of the present study; therefore, it is unlikely that the hit sequences showing less similarity than that of *O. sativa* (max score: 45; minimum *E*-value: 2 e^−4^) exhibited *ortho*-hydroxylation of cinnamates to form simple coumarins. However, the boundary line dividing the *ortho-hydroxylase* sequence involved in simple coumarin biosynthesis and the other 2OGDs remains unclear. *Liriodendron tulipifera*, a Magnoliales plant that arose from a more basal lineage than monocots, accumulates scopoletin (**4**) (Kang et al., 2014). *Cinnamomum cassia*, which is Laureales plant, also contains coumarin (**1**) (Choi et al., 2001). However, no significant similarity in the C-terminal sequence of AtF6′H1 was observed by TBLASTN search for ESTs in Magnoliales and Laurales plants. An unknown biosynthetic pathway of simple coumarins without 2OGD enzymes perhaps exists in plants.

Candidates of *ortho*-hydroxylases are mainly distributed in dicots, indicating that the biosynthesis of simple coumarins is a newer pathway of plant secondary metabolism, compared to flavonoids, which extensively occur in the plant kingdom (Harborne and Baxter, 1999; Williams and Grayer, 2004). Furthermore, biosynthetic pathways comprising apparently different enzyme sets evolutionally converged to form the coumarin core structure. Further analysis of plant *ortho*-hydroxylases at the molecular level would provide more details on the evolution of plant coumarins.

### Conflict of interest statement

The author declares that the research was conducted in the absence of any commercial or financial relationships that could be construed as a potential conflict of interest.

## Supplementary material

Supplementary Material 1 **The C-terminal sequences of the *ortho*-hydroxylases involved in biosynthesis of simple coumarins**.

Supplementary Material 2 **Results of TBLASTN search in EST sequences**.

Supplementary Material 3 **FASTA file of the protein sequences**.
