# Supplementary figures and images for: 2-Oxoglutarate-dependent dioxygenases in the biosynthesis of simple coumarins
Source: Front Plant Sci. 2014 Nov 3;5:549. doi: 10.3389/fpls.2014.00549 (PMC4217350; doi:10.3389/fpls.2014.00549)

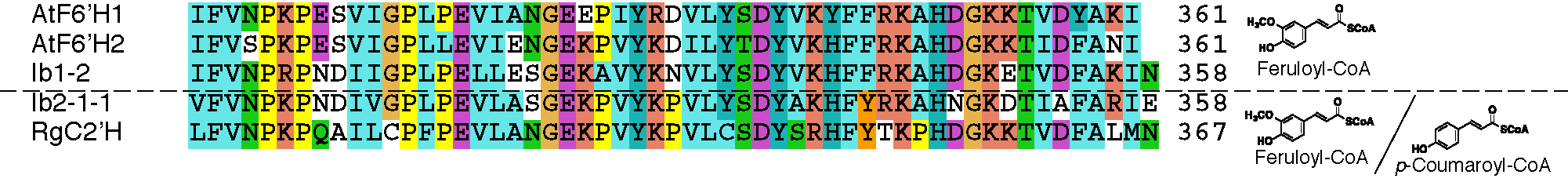

Supplement: Supplementary Material 2 — Results of TBLASTN search in EST sequences. [file Image1.TIF]
